# Decay of Kelvin‐Helmholtz Vortices at the Earth's Magnetopause Under Pure Southward IMF Conditions

**DOI:** 10.1029/2020GL087574

**Published:** 2020-06-29

**Authors:** T. K. M. Nakamura, F. Plaschke, H. Hasegawa, Y.‐H. Liu, K.‐J. Hwang, K. A. Blasl, R. Nakamura

**Affiliations:** ^1^ Space Research Institute Austrian Academy of Sciences Graz Austria; ^2^ Institute of Space and Astronautical Science Japan Aerospace Exploration Agency Sagamihara Japan; ^3^ Department of Physics and Astronomy Dartmouth College Hanover NH USA; ^4^ Southwest Research Institute San Antonio TX USA; ^5^ Institute of Physics University of Graz Graz Austria

**Keywords:** Kelvin‐Helmholtz instability, magnetic reconnection, magnetopause, southward IMF, PIC simulation, turbulence

## Abstract

At the Earth's low‐latitude magnetopause, clear signatures of the Kelvin‐Helmholtz (KH) waves have been frequently observed during periods of the northward interplanetary magnetic field (IMF), whereas these signatures have been much less frequently observed during the southward IMF. Here, we performed the first 3‐D fully kinetic simulation of the magnetopause KH instability under the southward IMF condition. The simulation demonstrates that fast magnetic reconnection is induced at multiple locations along the vortex edge in an early nonlinear growth phase of the instability. The reconnection outflow jets significantly disrupt the flow of the nonlinear KH vortex, while the disrupted turbulent flow strongly bends and twists the reconnected field lines. The resulting coupling of the complex field and flow patterns within the magnetopause boundary layer leads to a quick decay of the vortex structure, which may explain the difference in the observation probability of KH waves between northward and southward IMF conditions.

## Key Points

- Three‐dimensional fully kinetic simulation of Kelvin‐Helmholtz instability at the Earth's magnetopause under the southward IMF condition is performed
- Fast reconnection causes a rapid decay of the nonlinear vortex structure in the early nonlinear growth phase of the instability
- The vortex decay can lead to a lower probability of observing magnetopause Kelvin‐Helmholtz waves/vortices during southward IMF periods

## 1 Introduction

When the magnetic field is oriented nearly perpendicular to the direction of the plasma shear flow, the flow easily satisfies the super‐Alfvénic unstable condition for the Kelvin‐Helmholtz instability (KHI) (Chandrasekhar, 1961). When the interplanetary magnetic field (IMF) is strongly northward or southward, this configuration is realized at the Earth's low‐latitude magnetopause across which the velocity shear between the magnetosheath and magnetosphere persistently exists. Indeed, clear signatures of surface waves and nonlinear flow vortices, which could be generated by the KHI, have been frequently observed during periods of the northward IMF (e.g., Fairfield et al., 2000; Foullon et al., 2008; Hasegawa et al., 2004, 2006; Kavosi & Raeder, 2015; Kivelson & Chen, 1995; Kokubun et al., 1994; Moore et al., 2016; Sckopke et al., 1981; Slinker et al., 2003). Given a number of past theoretical and numerical studies suggesting that the KHI causes effective mass, momentum, and energy transfer across the boundary, this process has been believed to effectively contribute to forming the Earth's low‐latitude boundary layer (LLBL), where plasmas of magnetosheath and magnetospheric origins are mixed, at least during the northward IMF periods (e.g., Nakamura, 2020, and references therein).

On the other hand, the signatures of the magnetopause KH waves have been much less frequently observed during periods of the southward IMF (e.g., Kavosi & Raeder, 2015). Whereas the nonlinearly developed KH vortices have frequently been observed at the low‐latitude magnetopause during the northward IMF periods (e.g., Hasegawa et al., 2006), only a few observation events have been reported on the non‐linear evolution of the magnetopause KHI during the southward IMF periods (e.g., Hwang et al., 2011). Although these past observations showed a clear difference in the observation probability of the KH waves/vortices between northward and southward IMFs, the reason that this difference occurs is still not fully understood.

Theoretically, in the ideal magnetohydrodynamics (MHD) regime, the magnetopause boundary layer can be unstable for the KHI even for the southward IMF as long as the magnetic field component parallel to the shear flow is weak enough (Miura & Pritchett, 1982). Indeed, past global MHD simulations of the magnetosphere (Claudepierre et al., 2008) and local 3‐D MHD simulations of the low‐latitude magnetopause (e.g., Brackbill & Knoll, 2001) under the southward IMF conditions demonstrated clear evolutions of the KH waves and vortices along the low‐latitude magnetopause. Based on the MHD and Hall‐MHD simulations under southward IMF conditions, Ma et al. (2014) recently showed that patchy magnetic reconnection, with a typical fast rate on the order of 0.1 (e.g., Liu et al., 2017), is induced by the flow of the nonlinear KH vortices and generates complex flux ropes within the vortices. In this study, we performed the first 3‐D fully kinetic simulation, which fully resolves the reconnection structure from the electron kinetic to MHD scales, of the KHI induced at the magnetopause under the southward IMF condition. The simulation demonstrates that the fast reconnection occurs at multiple locations along the edge of the vortex and leads to a quick decay of the vortex structures. These results may explain the difference of the observation probability of the magnetopause KH waves/vortices between northward and southward IMFs.

## 2 Simulation Settings

We performed a 3‐D simulation of the low‐latitude magnetopause under the pure southward IMF condition, using the fully kinetic particle‐in‐cell code VPIC (Bowers et al., 2008, 2009). The initial simulation settings are obtained by modifying the settings employed in Nakamura, Hasegawa et al. (2017) and Nakamura, Eriksson et al. (2017), which modeled an Magnetospheric Multiscale (MMS) observation event of the magnetopause KH waves under a northward IMF condition. In Nakamura, Hasegawa et al. (2017) and Nakamura, Eriksson et al. (2017), the initial density, magnetic field, and ion bulk velocities across the magnetopause were set to the values obtained from the MMS observations. In this study, the magnetic field is modified to be oriented in the purely southward (−*z*) and northward (+*z*) on the magnetosheath and magnetospheric sides, respectively, by setting a Harris‐type current sheet *B*_*z*_(*y*) = B_0_tanh(*y*/*D*_0_), where *D*_0_ = 3.33d_i_ is the initial half thickness of the shear layer and *d*_i_ = c/ω_pi_ is the ion inertial length based on the Harris density component n_0_. The initial density ratio and the amplitude of the shear flow are set up to be *n*_2_/*n*_1_ = 0.3 and |*V*_0_| = 1.1*V*_A_ based on *n*_1_ and *B*_0_, respectively. Here *n*_1_ = *n*_0_ and *n*_2_ are the initial densities on the magnetosheath and magnetospheric sides, respectively. The electron temperature is set as uniform, while the ion temperature is set to satisfy pressure balance, where the ion‐to‐electron temperature ratio in the magnetosheath is set to be *T*_i1_/*T*_e0_ = 3.0. The ion‐to‐electron mass ratio is m_i_/m_e_ = 25, and the ratio between the electron plasma frequency and the gyrofrequency based on *n*_0_ and *B*_0_ is ω_pe_/Ω_e_ = 1.0. The system size is *L*_*x*_ × L_y_ × L_z_ = 50d_i_ × 50d_i_ × 50d_i_ = 1,024^3^ cells with a total of 2.2 × 10^11^ superparticles. The system length (*L*_*x*_) corresponds to the wavelength of the theoretical fastest growing KH mode *λ*_KH_ (Miura & Pritchett, 1982). The system is periodic in *x* and *z*, and *y* boundaries are modeled as perfect conductors for the fields and reflecting for the particles. In these settings, KH vortex is expected on the *x*‐*y* plane, while reconnection geometry is expected on the *y*‐*z* plane.

## 3 Results

### 3.1 Overview of the Simulation Results

**Figure 1:**
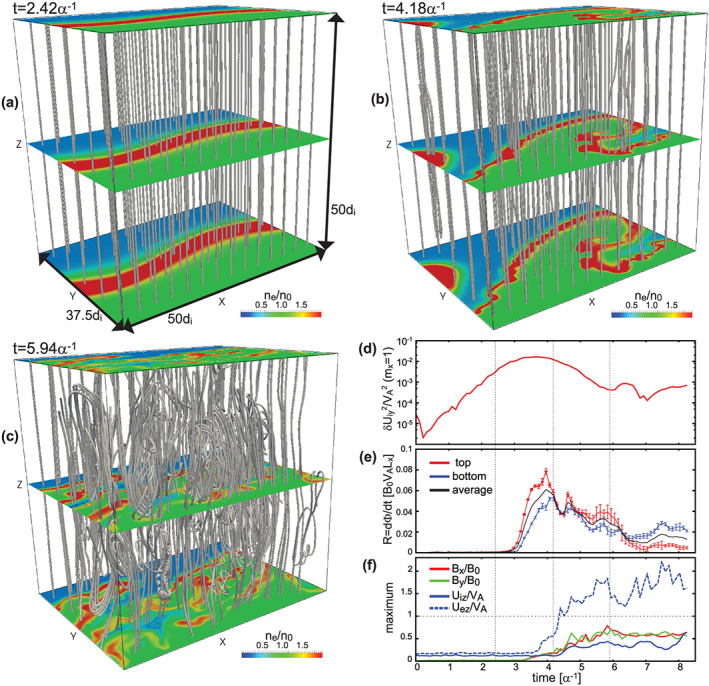
(a–c) Three‐dimensional views at (a) *t* = 2.42*α* ^−1^, (b) *t* = 4.18*α* ^−1^, and (c) *t* = 5.94*α* ^−1^ of selected magnetic field lines whose foot points are uniformly distributed in the simulation domain with contours in the *x*‐*y* plane at *z* = 0, L_z_/2 and L_z_ of n_e_. (d–f) Time evolutions of (d) δU_iy_^2^ for *m*_*x*_ = 1 KH mode, (e) the normalized, global reconnection rate R computed from the integrated magnetic flux that crosses the separatrix (mixing) surfaces, defined as |F_e_| = 0.99, on the top (red) and bottom (blue) sides, and (f) maximum values of B_x_, B_y_, U_iz_ and U_ez_ components. Here, F_e_ = (n_e1_ − n_e2_)/(n_e1_ + n_e2_) is the mixing fraction for electrons where n_e1_ and n_e2_ are the densities of electrons originally located in the bottom and top regions, respectively.

Figures 1a–1c show the time evolution of 3‐D views of selected magnetic field lines and electron density contours in the *x*‐*y* plane at *z* = 0, *L*_*z*_/2 and *L*_*z*_ from the linear (*t* < 3*α* ^−1^) to the early nonlinear (*t* ~ 4–6*α* ^−1^) growth phases of the KHI. Here *α* ^−1^ = *λ*_KH_/*V*_0_ is the time unit for the growth phase of the KHI (Nakamura et al., 2013). Figure 1d shows the time evolution of *δ*U_iy_^2^ for the *m*_*x*_ = 1 mode indicating the growth of the KHI. The vertical lines in Figure 1d correspond to the times shown in Figures 1a–1c. The results show that magnetopause surface waves are formed by the KHI even in the case of the pure southward IMF on a similar time scale to the northward IMF cases (Nakamura et al., 2013; Nakamura, Eriksson et al., 2017) in which the saturation of the linear growth starts at around *t* ~ 3*α*^−1^ (Figure 1a). In the early nonlinear growth phase, the thin compressed layer is formed along the edge of the vortex (Figure 1b). The compressed layer fluctuates as the field lines rooted at the layer are disturbed (Figure 1b), which results from the evolution of magnetic reconnection at the layer as will be shown in section 3.2. After the onset of reconnection, the vortex structure quickly decays only in 1–2*α* ^−1^, accompanied by the formation of complex field line structures in the decayed layer (Figure 1c).

Figure 1e shows the time evolution of the normalized, global reconnection rate *R* computed from the integrated magnetic flux that crosses the separatrix (mixing) surfaces on the top (red) and bottom (blue) sides (see Daughton et al., 2014, for details of the definition of *R*). Reconnection starts soon after the linear growth of the KHI ends (*t* ~ 3*α* ^−1^). The peak reconnection rate (*R* ~ 0.06–0.08), which is renormalized as 0.08–0.11 when considering the local density (*n*–*n*_1_ + *n*_0_–2*n*_0_) near the current sheet center, is close to the typical rate of fast reconnection (e.g., Liu et al., 2017) and similar to the values seen in past MHD and Hall‐MHD simulations for the southward IMF (Ma et al., 2014). Simultaneously with the onset of reconnection, the in‐plane magnetic field components (*B*_*x*_ and *B*_*y*_), which correspond to the newly reconnected field, start increasing and grow to significant amplitudes ~0.5B_0_ (see red and green curves in Figure 1f). Together with the evolution of the reconnected field, the *z* component of the ion and electron bulk velocities (*U*_iz_ and *U*_ez_) also increases (see solid and dashed blue curves in Figure 1f), corresponding to the evolution of ion and electron outflow jets. The peak *U*_iz_ and *U*_ez_ values are about 0.5*V*_A_ and 2*V*_A_ (~0.7*V*_A_ and 3*V*_A_ when considering the local density), respectively, indicating that reconnection well matures within the vortex layer.

### 3.2 Local Reconnection Structures

**Figure 2:**
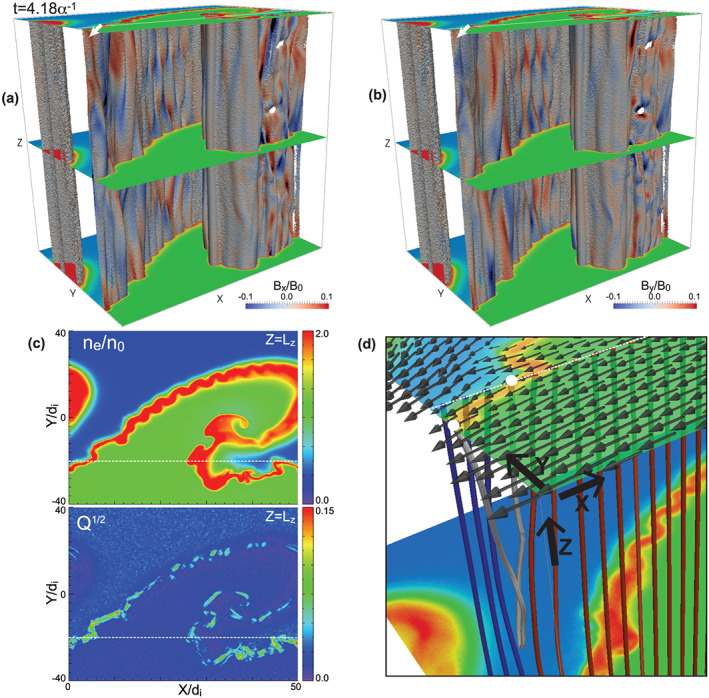
Signatures of reconnection at t = 4.18*α* ^−1^. (a, b) Three‐dimensional views of contour surfaces of n_e_ = 1.9n_0_ colored by (a) B_x_ and (b) B_y_ with n_e_ contours in the *x*‐*y* plane at z = 0, L_z_/2 and L_z_. (c) Two‐dimensional contours in the *x*‐*y* plane at *z* = *L*_*z*_ of n_e_ and $\sqrt{Q}$, where *Q* is the measure of agyrotropy (Swisdak, 2016). (d) Zoomed‐in‐view near the region marked by the white arrow in Figures 2a and 2b of the ion flow vectors on the *x*‐*y* plane at *z* = *L*_*z*_ and selected magnetic field lines traced from the white dotted line locating at *y* = −10.1d_i_ and *z* = *L*_*z*_ (the same line in Figure 2c). The red, blue, and gray field lines are the ones whose foot points on the white dotted line locate in the magnetosheath, magnetosphere, and boundary layer regions, respectively. The white circles in Figure 2d show the foot points on the *x*‐*y* plane at *z* = *L*_*z*_ of the gray field line.

Figures 2a and 2b show 3‐D views at *t* = 4.18*α* ^−1^ (the same time as Figure 1b) of contour surfaces of *n*_e_ = 1.9*n*_0_ locating near the center of the compressed current layer, colored by *B*_*x*_ and *B*_*y*_, corresponding to the reconnected field. Patterns of negative and positive peaks of these components are seen at multiple locations, indicating that reconnection occurs at multiple points along the compressed layer. Note that the *B*_*x*_ and *B*_*y*_ components are initially set to be 0 in the present simulation setting, and these components do not evolve exceeding the noise level of the PIC simulation in the corresponding 2‐D simulation in the *x*‐*y* plane (the vortex plane) in which reconnection does not occur (not shown). Figure 2c shows 2‐D contours in the *x*‐*y* plane at *z* = *L*_*z*_ of *n*_e_ and $\sqrt{Q}$, where *Q* = (*P*_*exy*_^2^ + *P*_*exz*_^2^ + *P*_*eyz*_^2^)/(*P*_*e*⊥_^2^ + 2*P*_*e*⊥_*P*_*e*∥_) is the measure of agyrotropy proposed in Swisdak (2016). This measure is known to be enhanced within the reconnection layer such as near the X‐lines and separatrices. Significant enhancement of Q seen along the compressed layer also indicates the occurrence of reconnection along the layer.

Here we focus on a location marked by the white arrow in Figures 2a and 2b, where the positive *B*_*y*_ variation (Figure 2b) is seen, which is a typical pattern of the north side of the reconnected field lines connecting the negative *B*_*z*_ field in the magnetosheath and the positive *B*_*z*_ field in the magnetosphere. Notice that in this location, the positive *B*_*x*_ variation is also seen in addition to the positive *B*_*y*_ variation. This is because the local shear flow twists the reconnected field lines in the shear flow direction (nearly the −*x* direction), as seen in a recent 3‐D fully kinetic simulation of reconnection with the local shear flow in the direction perpendicular to the reconnection plane (Liu et al., 2018). Figure 2d shows a zoomed‐in view near the region marked by the white arrow in Figures 2a and 2b of the magnetic field lines rooted on the white dotted line, and highlights one of these twisted reconnected field lines (see the gray‐colored field line in Figure 2d). The white circles in Figure 2d show the foot points of this reconnected field line in the *x*‐*y* plane at *z* = *L*_*z*_. Since the foot point on the magnetosheath side flows in the –*x* direction faster than the other foot point (see arrows near the foot points in Figure 2d), the former is shifted further in the –*x* direction, resulting in the reconnected field line being twisted in the –*x* direction. Thus, for the southward IMF case, the complex vortex motion, which produces local shear flows at multiple locations within the vortex layer, is largely inclined from the reconnection plane and can easily disturb the structure of the reconnected field lines.

### 3.3 Signatures of KH Wave and Vortex

**Figure 3:**
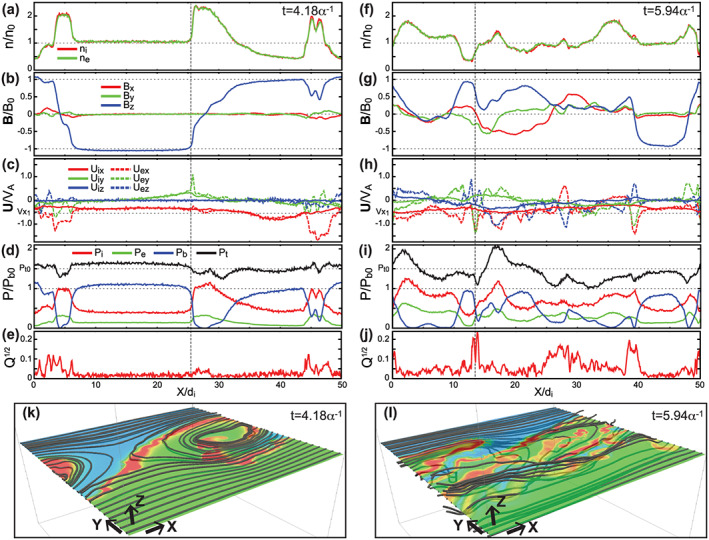
(a–j) Cuts at (a–e) *t* = 4.18*α* ^−1^ and (f–j) *t* = 5.94*α* ^−1^ along *x* at *y* = −10.1d_i_ and *z* = L_z_ (the same line as the white dotted line in Figures 2c and 2d) of (a, f) the density for ions and electrons n_i,e_, (b, g) the magnetic field **B**, (c, h) the bulk flow velocities for ions and electrons **U**_i,e_, (d, i) the plasma, magnetic and total pressures (P_i,e_, P_b_, and P_t_), and (e, j) $\sqrt{Q}$. The vertical lines indicate the locations of the positive *U*_iy_ peaks. (k, l) Ion streamlines at (k) *t* = 4.18*α* ^−1^and (l) *t* = 5.94*α* ^−1^ rooted at *x* = 0(=*L*_*x*_) and *z* = L_z_/2 with density contours in the *x*‐*y* plane at *z* = *L*_*z*_/2.

Figures 3a–3j show cuts at *t* = 4.18*α* ^−1^ (the same time as Figures 1b and 2) and *t* = 5.94*α* ^−1^ (the same time as Figure 1c) along *x* at *y* = −10.1d_i_ (the same line as the white dotted lines in Figures 2c and 2d) of the density, magnetic field, bulk flow velocity, pressure components, and $\sqrt{Q}$. At *t* = 4.18*α* ^−1^, periodic variations for one wavelength of the KHI (λ_KH_ = 50d_i_) are seen in all field and plasma components. In particular, the combination of the negative‐to‐positive *U*_iy_ variation with the higher total pressure *P*_t_ than the average and the subsequent positive‐to‐negative *U*_iy_ variation with the lower *P*_t_ (compare before and after the vertical line in Figures 3c and 3d) shows a typical variation pattern of the KH waves in which the pressure gradient force is balanced with the centrifugal force (e.g., Hasegawa, 2012). Furthermore, the flow faster than the background magnetosheath (|*U*_ix_| > |*V*_x1_|, where *V*_x1_ = −V_0_/2) in the low‐density interval near *x* ~ 38d_i_ (see Figures 3a and 3c) shows a typical signature of the nonlinear vortex called the low‐density‐faster‐than‐sheath (LDFTS) plasmas (e.g., Hasegawa et al., 2006). Indeed, the ion streamlines at *t* = 4.18*α* ^−1^ (Figure 3k) show clear patterns of the rotating, non‐linear vortex flow in the *x*‐*y* plane. In addition to these signatures of the KH wave/vortex, reconnection signatures as discussed in section 3.2 are seen near the edge of the vortex (*x* < 5d_i_ and *x* > 43d_i_) where the finite *B*_*x*_, *B*_*y*_, *U*_iz_, and *U*_ez_ variations and the Q enhancements are seen with the sharp variations of the reconnecting field *B*_*z*_.

These reconnection signatures are quickly enhanced and mature in 1–2*α* ^−1^ as seen in Figure 1f and right panels in Figure 3. Figures 3g, 3h, and 3j show that the amplitudes of the reconnection parameters (*B*_*x*_, *B*_*y*_, *U*_iz_, *U*_ez_, and *Q*) at *t* = 5.94*α* ^−1^ become significantly larger than those at *t* = 4.18*α* ^−1^. Note that the *U*_iz_ variations (corresponding to ion outflow jets) are still rather weak at *t* = 4.18*α* ^−1^, while the variations become visibly larger at *t* = 5.94*α* ^−1^, indicating that the structures in the reconnection regions, which are initially formed in electron‐scales, evolve into ion scales as also seen in recent 3‐D simulations for the northward IMF (Nakamura, Eriksson et al., 2017; Nakamura, Hasegawa et al., 2017). On the other hand, as the reconnection parameters are enhanced, the signatures of the KH wave and vortex are rapidly disappearing; as seen in Figure 1d, the amplitude of the *m*_*x*_ = 1 mode is rapidly suppressed after the peak at *t* ~ 3.5*α* ^−1^ and falls down to the level nearly 2 orders of magnitude smaller than the peak value until *t* = 5.94*α* ^−1^. The cuts at *t* = 5.94*α* ^−1^ in Figure 3 show no clear periodic variations for λ_KH_ in any field and plasma components. In addition, the pattern of the *U*_iy_ variations no longer correlates with the pattern of the *P*_t_ variations (Figures 3h and 3i). Furthermore, the flow in the low‐density interval at *t* = 5.94*α* ^−1^ is also no longer faster than the magnetosheath plasma (Figures 3f and 3h at *x* ~ 12d_i_), indicating that the structure of the nonlinear vortex has decayed in this phase. Indeed, the ion streamlines at *t* = 5.94*α* ^−1^ (Figure 3l) no longer show typical patterns of vortex motions in the *x*‐*y* plane.

**Figure 4:**
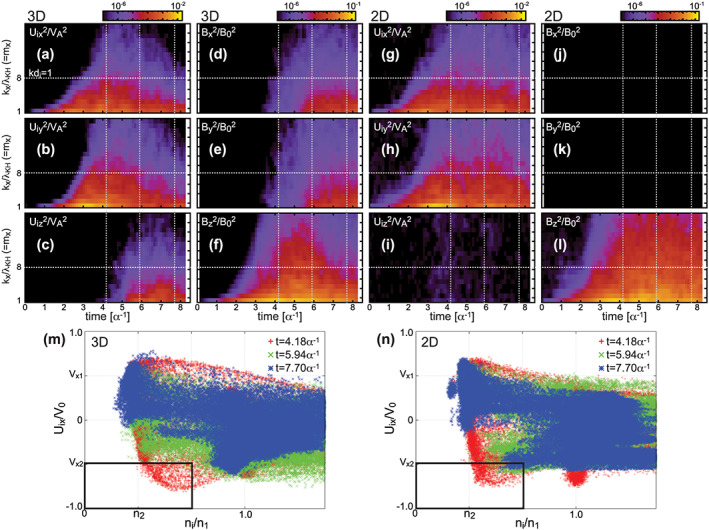
Results from (a–f) the 3‐D simulation and (g–l) the corresponding 2‐D simulation in the *x*‐*y* plane of (a–l) time evolutions of integrated squared amplitudes of k_x_ modes for *U*_ix_, *U*_iy_, *U*_iz_, *B*_*x*_, *B*_*y*_, and *B*_*z*_ in the range −6.25d_i_ < *y* < 6.25d_i_, and (m, n) scatter plots of *U*_ix_ versus *n*_i_ at *t* = 4.18*α* ^−1^, *t* = 5.94*α* ^−1^, and *t* = 7.70*α* ^−1^. The horizontal and vertical dotted lines in Figures 4a–4l indicate kd_i_ = 1 and the times shown in Figures 4m and 4n, respectively.

To extract the effects of reconnection in the decay process of the KH wave/vortex, we compared the present 3‐D simulation with the corresponding 2‐D simulation performed in the x‐y plane with the same setting as the 3‐D simulation. Figures 4a–4l show time evolutions of 1‐D spectra (*k*_*x*_) of three components of **U**_i_ and **B** in the 3‐D and 2‐D simulations. The results are similar in both 3‐D and 2‐D before the early nonlinear growth phase of the KHI (*t* < 3–4*α* ^−1^); the fastest‐growing KH mode (*m*_*x*_ = 1), which is seen for *U*_ix_, *U*_iy_, and *B*_*z*_, grows dominantly in the linear phase (*t* < 2*α* ^−1^), and subsequently, smaller‐scale modes of these parameters are enhanced during *t* ~ 2 to 3*α* ^−1^, corresponding to the formation of the thin compressed layers below the ion scale (*k*d_i_ > 1). After that, while in 2‐D the powers of the KH wave related modes (*U*_ix_, *U*_iy_, and B_z_) are only slightly suppressed at both large and small scales, in 3‐D these modes are considerably suppressed as the *U*_iz_, *B*_*x*_, and *B*_*y*_ modes, which are induced by reconnection, are enhanced. This occurs because the vortex flow in the *x*‐*y* plane is interrupted at multiple locations by flows in multiple reconnection regions, leading to the turbulent decay of the vortex motion in the *x*‐*y* plane simultaneously accompanied by the turbulent deformation of the reconnected field lines by the disrupted flow. As a result, at *t* = 5.94*α* ^−1^ a large part of the streamlines within the vortex layer are complicatedly bent in the *z* direction as seen in Figure 3l, and simultaneously, the reconnected field lines are largely disturbed within the layer as seen in Figure 1c. The rapid decay of the vortex motion by reconnection also leads to a rapid reduction of the LDFTS plasmas only in 3‐D as shown in Figures 4m and 4n; in 3‐D the LDFTS plasmas present in the region marked by the black box almost disappear after *t* > 5–6*α* ^−1^, while in 2‐D there are still some counts in the region even after *t* > 7*α* ^−1^.

## 4 Summary and Discussion

We performed the first 3‐D fully kinetic simulation of the KHI at the magnetopause under the pure southward IMF condition. The simulation demonstrates that magnetic reconnection, with a typical fast rate on the order of 0.1, is induced at multiple locations along the vortex edge in an early nonlinear growth phase of the KHI. The reconnection outflow jets, which grow in the direction nearly perpendicular to the initial shear flow, significantly disrupt the flow of the nonlinear KH vortex. On the other hand, the disrupted vortex flow complicatedly bends and twists the reconnected field lines toward the direction out of the reconnection plane. The resulting coupling of the complex field and flow patterns within the magnetopause boundary layer leads to a quick decay of the vortex structure. These simulation results suggest that clear signatures of the KH waves/vortices may be observed only for a limited phase during periods of the southward IMF, and this may explain the low observation probability of KH waves/vortices during periods of the southward IMF (e.g., Kavosi & Raeder, 2015).

For the wave signatures, the present results predict that the wave structures can be visibly identified only during the late linear growth phase through the early nonlinear growth phase of the KHI. In the Earth's magnetosphere, this wave‐expected interval would correspond to the prenoon or postnoon low‐latitude magnetopause to the flank regions if the KHI is initially induced at a prenoon or postnoon region. Note that during the southward IMF periods, other processes such as reconnection that is not induced by the KH waves (e.g., Burch, Torbert et al., 2016; Phan et al., 2000; Trattner et al., 2007) and flux transfer events (FTEs) (e.g., Dunlop et al., 2005; Russell & Elphic, 1978) are also expected to disturb the dayside, low‐latitude magnetopause. Although these processes may also hinder the growth of the KH waves at the low‐latitude magnetopause, the self‐decay process of the KH waves/vortices shown in this paper would also significantly reduce the observation probability of the KH waves/vortices.

For the nonlinear vortex signatures, the present 3‐D simulation shows that the nonlinear vortex signatures such as the LDFTS plasmas are seen for only about 1*α* ^−1^ (=λ_KH_/*V*_0_) in the early non‐linear growth phase of the KHI (*t* ~ 4–5*α* ^−1^). In the Earth's magnetosphere, this vortex‐expected interval would correspond to a part of the magnetopause with its length close to λ_KH_ (approximately a few Earth radii) if the KHI propagates along the magnetopause at the phase speed close to *V*_0_. Note that based on a 3‐D fully kinetic simulation of the KHI under the northward IMF conditions, Nakamura, Eriksson et al. (2017) showed that the vortex‐induced reconnection (VIR) also disturbs the vortex structure and reduces the LDFTS plasmas even in the northward IMF case. However, this happens much later in the northward IMF case (*t* ~ 7*α* ^−1^) than in the present southward IMF case. This is because for the northward IMF, the magnetic shear across the magnetopause and the related antiparallel component of the magnetic field (i.e., the reconnecting field), which controls the maximum speed of the reconnection jets, are much weaker than for the southward IMF case. These simulation results naturally predict that the observation probability of the nonlinear KH vortex for the southward IMF would be much lower than for the northward IMF.

Hwang et al. (2011) reported an event in which Cluster observed the nonlinear vortex structures that can be interpreted as being formed by the KHI during the southward IMF periods. This event features the structure of the observed nonlinear vortices being irregular and temporally intermittent, indicating that the structure of the vortices was being disturbed during this event. This point may be related to the decay process of the nonlinear KH vortex seen in the present simulation. Although it is difficult to resolve the thin vortex edge layers and confirm the VIR structures in the reported Cluster event, the high‐time‐resolution fields and plasma data by the MMS mission (Burch, Moore et al., 2016) would be useful to analyze such small‐scale VIR physics for the southward IMF as performed on a magnetopause VIR event for the northward IMF (Eriksson et al., 2016, 2017; Li et al., 2016; Nakamura, Hasegawa et al., 2017; Nakamura, Eriksson et al., 2017; Vernisse et al., 2016; Stawarz et al., 2016).

Note that this study treats only a condition in which the Alfvén Mach number of the initial shear flow is close to unity (*M*_A_ = *V*_0_/*V*_A_ = 1.1). In such a strong shear flow case, the reconnected structures such as flux ropes propagate in directions largely inclined to the background flow. However, for smaller M_A_, which would be realized at regions closer to the subsolar point, the structures would propagate faster in the outflow direction, which may allow to more easily observe characteristic reconnection signatures such as the FTE‐like positive‐negative variations of the normal field, as predicted by past 3‐D MHD simulations (Brackbill & Knoll, 2001; Knoll & Brackbill, 2002). Note also that although this study treats a Harris‐type initial equilibrium condition as done in some past simulation studies of the magnetopause (e.g., Daughton et al., 2014), past observations showed various types of the magnetopause current sheets like the force‐free sheets (e.g., Panov et al., 2011). Performing additional runs with different parameters and initial equilibriums would be a necessary next step to more comprehensively understand the realistic roles, states, and properties of the KHI under the southward IMF conditions.

## Data Availability

The simulation data are available online (via http://doi.org/10.5281/zenodo.3676527).
